# Social Network Characteristics and Depressive Symptoms of Italian Public Health Medical Residents: The Public Health Residents’ Anonymous Survey in Italy (PHRASI)

**DOI:** 10.3390/bs13110881

**Published:** 2023-10-25

**Authors:** Lorenzo Stacchini, Alessandro Catalini, Valentina De Nicolò, Claudia Cosma, Veronica Gallinoro, Angela Ancona, Nausicaa Berselli, Marta Caminiti, Clara Mazza, Giuseppa Minutolo, Fabrizio Cedrone, Vincenza Gianfredi, on Behalf of the Working Group on Public Mental Health 2021/2022 of the Medical Residents’ Assembly of the Italian Society of Hygiene and Preventive Medicine

**Affiliations:** 1 Department of Health Sciences, University of Florence, 50121 Florence, Italyveronica.gallinoro@unifi.it (V.G.); 2 Department of Biomedical Sciences and Public Health, Università Politecnica delle Marche, 60100 Ancona, Italy; 3 Department of Public Health and Infectious Disease, Sapienza University of Rome, 00185 Rome, Italy; 4 School of Hygiene and Preventive Medicine, Vita-Salute San Raffaele University, 20132 Milan, Italy; ancona.angela@hsr.it; 5 Department of Biomedical, Metabolic and Neural Sciences, University of Modena and Reggio Emilia, 41125 Modena, Italy; nausicaa.berselli@unimore.it; 6 Department of Medicine and Surgery—Sector of Public Health, University of Perugia, 06100 Perugia, Italy; 7 Department of Public Health, Experimental and Forensic Medicine, University of Pavia, 27100 Pavia, Italy; 8 Department of Health Promotion, Mother and Child Care, Internal Medicine and Medical Specialties, University of Palermo, 90127 Palermo, Italy; 9 Hospital Management, Local Health Authority of Pescara, 65100 Pescara, Italy; fabrizio.cedrone@asl.pe.it; 10 Department of Biomedical Sciences for Health, University of Milan, 20133 Milan, Italy; vincenza.gianfredi@unimi.it

**Keywords:** cross-sectional study, depression, healthcare workers, mental health, public health, social networking

## Abstract

Despite the high impact of the COVID-19 pandemic on social interactions and healthcare workers’ (HWs’) mental health, few studies have investigated the association between social network characteristics and HWs’ mental health, particularly during the pandemic. Therefore, we aimed to assess the associations between public health residents’ (PHRs’) social network characteristics and depression. We used data from the Public Health Residents’ Anonymous Survey in Italy (PHRASI), a nationwide cross-sectional study. Social network characteristics were self-reported. Depressive symptoms were assessed using the nine-item Patient Health Questionnaire. Linear and logistic models adjusted for age, sex, physical activity, and alcohol were used. A moderation analysis by sex was also performed. A total of 379 PHRs participated in the survey (58% females, median age of 30 years). More peer-to-peer (odds ratio OR = 0.62 (0.47–0.83)) and supervisor support (OR = 0.49 (0.36–0.68)), more social participation ((OR) = 0.36 95% CI (0.25–0.50)), and having a partner (OR = 0.49 (0.25–0.96)) were significantly associated with a lower risk of clinically relevant depressive symptoms. Work-to-private-life interference was significantly associated with a higher risk of clinically relevant depressive symptoms (OR = 1.77 (1.28–2.45)). Promoting a supportive work environment and social participation as well as reducing work-to-private life interference can contribute to reducing the high burden among PHRs.

## 1. Introduction

Depression is one of the most prevalent mental illnesses, affecting 3.8% of the global population and resulting in approximately 280 million people suffering from depression [1,2]. Depression is a remittent disease that represents a main determinant of years lived with disability [3], an excess of mortality, and a reduction in life expectancy (14 years in males and 10 years in females) [4,5]. Because of the high burden of the disease and the associated healthcare costs, the World Health Organization (WHO) defines depression as a major public health problem [6]. Moreover, the WHO’s projections estimate that depression will increase, becoming the leading cause of burden in 2030 [1,7].

Despite efforts, the causes of depression are not completely known, but there is recognition of a multifactorial etiology, including genetic, environmental, biological, psychosocial, and social components [8]. Among them, the rapid spread of the COVID-19 pandemic greatly impacted mental health, leading to a stark rise in depressive disorders, with an estimated increase of 27.6% [range 25.1–30.3%] and an additional 53.2 million [95% uncertainty interval 44.8–62.9] cases of depression worldwide [9]. Generally, restrictive measures put in place to stop the spread of COVID-19 infection played an important role in dramatically increasing the overall number of depression cases [10]. In more detail, quarantine and social distancing further increased the risk of social isolation, loneliness, and depression [11,12], highlighting how social isolation is strictly linked to physical [13] and mental health [14,15,16,17,18]. Regarding mental health, various studies [19,20,21,22] have shown that social networks, i.e., systems of social relationships in which individuals embed [23,24], are related to depressive disorder. They particularly pointed out the association between limited support networks and lower psychological well-being [18,25,26] and the prominent role of a supportive network in providing psychological advantages and reducing mental distress [27]. Furthermore, evidence highlights that low social integration, i.e., individual participation in social relationships and engagement in social activities [28], is associated with poor mental health outcomes [29].

On the contrary, the COVID-19 pandemic led to unprecedented restrictions on social life and drastically impacted social integration and its protective role in mental well-being [30]. According to a recent meta-analysis (2021), the social impact of the COVID-19 pandemic imposed a huge mental health burden on society, especially on quarantined people, COVID-19 patients, and healthcare workers compared with the general population [31]. Despite the high impact of the pandemic on healthcare workers’ mental health, few studies have been conducted to investigate how social networks and social support could influence healthcare workers’ mental health, particularly during the COVID-19 pandemic. Moreover, previous studies have mainly focused on structured healthcare workers, and no research has been conducted during the years of residency, particularly among public health residents (PHRs).

In order to bridge this gap in knowledge, the current nationwide, cross-sectional study aimed to assess the associations between PHRs’ social network characteristics and depression. We hypothesized that a low and not supportive social network might be associated with an increased risk of depression during residency. Lastly, we performed a moderation analysis by sex to examine if the effect of social network characteristics on depressive symptoms is the same across genders.

## 2. Materials and Methods

### 2.1. Study Population and Design

This study originated from the Public Health Residents’ Anonymous Survey in Italy (PHRASI), a nationwide, cross-sectional investigation aimed at assessing the mental health of Italian Public Health Medical Residents and its determinants.

PHRASI comprises an 88-item online questionnaire, which includes socio-demographic questions and tools for evaluating various aspects of mental health. All the questionnaires used in the survey were drawn from the existing literature and had been previously validated. To ensure internal consistency, we calculated the Cronbach’s α values for each of the adopted tools (Supplementary Table S1). The study protocol was published previously [32]. In brief, the administration of the survey using Google Forms was made possible through the robust network of the Medical Residents’ Assembly of the Italian Society of Hygiene and Preventive Medicine. The survey link was distributed to all Italian postgraduate Public Health school residents via a mailing list. Furthermore, to enhance participation, researchers directly contacted each representative to encourage the distribution of the survey among their colleagues. Participation was voluntary and anonymous, and no incentives were provided for participating. Data collection began on 14 June 2022 and finished on 26 July 2022. A sample size of 314 was calculated using the Charan and Biswas formula [33], taking into account the expected lifetime prevalence of mental health disorders (28.5%) [34] and the expected error of the confidence interval estimate (5.0%).

### 2.2. Social Network Characteristics

Both functional and structural characteristics of the social network were assessed. Regarding functional characteristics of the social network, peer-to-peer support, supervisor support, and work-to-private life interference were measured, using Likert scale questions as follows: “I can rely on the help of my colleagues” (1—Never; 5—Always), “I can rely on the help of my boss” (1—Never; 5—Always), “My work often interferes with my family, social or personal duties” (1—Always; 5—Never) [35].

For the structural characteristics of the social network, the distance between the residential and working regions, the number of family members, cohabitation, having a partner, and social participation were assessed. The distance between the residential and working regions was determined by measuring the distance between the geographical center (centroid) of each region and the centroid of the other region using the Vincenty method [36]. The number of family members was calculated by taking into account cohabitation, having a partner, and the number of children. Cohabitation refers to living with someone (e.g., a flatmate or partner) in the same household. Having a partner was assessed by inquiring whether the participant was in a stable relationship (yes/no). Social participation was assessed using the 5th item of the WHO-5 Well-being questionnaire [37], which asks, “My daily life has been filled with things that interest me”. The WHO-5 Well-being is a validated questionnaire based on a 5-item Likert scale (0—at no time; 5—all of the time = 5) [37].

### 2.3. Assessment of Depressive Symptoms

Prevalent depressive symptoms were assessed using a validated Italian version of the 9-item Patient Health Questionnaire (PHQ-9) [38]. This questionnaire consists of nine items rated on a four-point scale, ranging from 0 = “not at all” to 3 = “nearly every day”. The response options were utilized to compute a continuous total score, which ranges from 0 (indicating the absence of symptoms) to 27 (indicating the presence of all symptoms nearly every day). For the purpose of this study, two predefined cut-offs were applied to identify (i) the presence of mild to severe depressive symptoms, with a cut-off score equal to or greater than 5 [39], and (ii) clinically relevant depressive symptoms (moderate to severe), with a cut-off score equal to or greater than 10.

### 2.4. Covariates

The administered questionnaire allowed us to gather sociodemographic data, including gender and age. Additionally, lifestyle factors such as physical activity and alcohol consumption were also assessed. Alcohol consumption was evaluated using the Alcohol Use Disorders Identification Test (AUDIT-C) [40,41]. AUDIT-C comprises 3 questions, with each question having a scale ranging from 0 to 4, resulting in a total score ranging from 0 to 12. The total score was subsequently dichotomized into “high-risk drinking” (equal to or greater than 5 for males, and equal to or greater than 4 for females). Physical activity was measured using the International Physical Activity Questionnaire (IPAQ) [42] in Italian, provided by the Italian Society of Endocrinology (ISE). The metabolic equivalent task was calculated following ISE indication. Subsequently, we categorized each participant into three groups based on IPAQ score: a total score of <700 was classified as “Insufficiently active”, a score between 700 and 2519 was classified as “Sufficiently active”, and a score exceeding 2519 was classified as “Active”.

### 2.5. Statistical Analysis

Continuous variables were provided as median and interquartile range, while categorical variables were described as frequencies and percentages. To examine the relationships between predictor variables, a correlation matrix was generated using Kendall’s tau correlation coefficient. Multivariate linear regression models, adjusted for sex and age, were performed with the PHQ-9 score as the continuous dependent variable (model 1). Additionally, multivariate logistic regressions were performed using the PHQ-9 score as a dichotomous dependent variable to assess the presence of depressive symptoms, yielding odd ratios (ORs) and 95% confidence intervals (95% CIs) (model 2). In this case, two pre-defined PHQ-9 cut-offs were considered. Furthermore, a sensitivity analysis was carried out in which the multivariate regression models were additionally adjusted for alcohol consumption (assessed using the AUDIT-C risk category) and physical activity (assessed using IPAQ activity category) (model 3). A moderation analysis was conducted by sex, with females as a reference, using multivariate logistic regressions (outcome: PHQ-9 ≥ 10) adjusted by age for each independent variable. An interaction term was created, and significant effects were identified when the predictor and the moderator significantly affected the outcome. In cases where the interaction term was not significant, the predictor OR applies to both males and females. However, when significant, the interaction term applies to males, and the predictor to females. Associations with *p* < 0.05 were considered statistically significant when using two-sided tests.

To assess multicollinearity, we examined Kendall’s tau correlation coefficient [43] between each independent variable, considering a strong correlation when Kendall’s tau correlation coefficient (τ) was equal to or greater than 0.50. All the analyses were performed using R 4.2.3.

### 2.6. Ethical Approval

This study did not require the approval of an ethics committee because the questionnaire data were anonymous, rendering it impossible to identify any respondent [44,45,46]. All data were entered into an anonymous password-protected computer database.

## 3. Results

### 3.1. Descriptive Characteristics of the Sample

The characteristics of the sample are summarized in Table 1. A total of 379 subjects participated to the Public Health Residents’ Anonymous Survey in Italy (PHRASI) study. The majority of young physicians who completed the questionnaire were females (58%). The median age of the sample was 30 years, with an interquartile range between 29 and 34. Most of the subjects had a partner (73%) and lived with others (74%). Mean, SD, skewness, and kurtosis are reported in Supplementary Table S2, and the statistical distribution is shown in Supplementary Figure S1. The correlation matrix between the study variables is reported in Supplementary Table S3.

Mild to severe depressive symptoms (PHQ-9 ≥ 5) were reported by 231 participants (59% females, 31 years old), whereas clinically relevant depressive symptoms (PHQ-9 ≥ 10) were reported by 97 participants (57% females, 31 years old).

### 3.2. Social Network Characteristics and Depressive Symptoms

Table 2 shows the association between functional and structural characteristics of social networks and depressive symptoms, as determined using the linear regression models adjusted for sex and age. Among functional characteristics of social networks, greater peer-to-peer support and supervisor support were associated with a lower PHQ-9 score. Specifically, for each increment in peer-to-peer support (β = −1.01 95% CI = (−1.52; −0.50)) and supervisor support (β = −1.20 95% CI = (−1.66; −0.74)), depressive symptoms decreased. Conversely, as the value related to work-to-private-life interference increased (β = 1.24 95% CI = (0.74; 1.74)), the PHQ-9 score increased.

Regarding structural characteristics of social networks, family members, having a partner, and social participation were significantly associated with PHQ-9. Specifically, for each unit increase in social participation, depressive symptoms decreased on a continuous scale (β = −2.13 95% CI = (−2.49; −1.76)). Having more family members was significantly associated with lower depressive symptoms (β = −0.81 95% CI = (−1.54; 0.08)). Similarly, having a partner is associated with fewer depressive symptoms (β = −1.42 95% CI = (−2.58; −0.25)).

### 3.3. Social Network Characteristics and Clinically Relevant Depressive Symptoms

Table 3 shows the results of the logistic regression adjusted for sex and age, with the PHQ-9 ≥ 10 score considered as a dichotomous dependent variable.

Among the functional characteristics of the social network, both peer-to-peer and supervisor support are significantly associated with a lower risk of clinically relevant depressive symptoms (OR = 0.72 (95% CI = 0.58; 0.90), OR = 0.69 (95% CI = 0.55; 0.85), respectively). Conversely, work-to-private-life interference is statistically significantly associated with a higher risk of clinically relevant depressive symptoms (OR = 1.59 (95% CI = 1.25; 2.01)).

Among structural characteristics of the social network, having a partner and social participation are significantly associated with a lower risk of clinically relevant depressive symptoms (OR = 0.59 (95% CI = 0.36; 0.97), OR = 0.39 (95% CI = 0.31; 0.50), respectively). The distance between residential and working regions, cohabitation, and the number of family members are not statistically significantly associated with clinically relevant depressive symptoms. Similar results were also obtained when considering PHQ-9 ≥ 5 (Supplementary Table S4).

In the model further adjusted for alcohol consumption and physical activity (sensitivity analysis), social participation, peer-to-peer, supervisor support, having a partner, and WLI remain significantly associated (OR = 0.36 (95% CI = 0.25; 0.50); OR = 0.62 (95% CI = 0.47–0.83); OR = 0.49 (95% CI = [0.36–0.68); OR = 0.49 (95% CI = 0.25–0.96); OR = 1.77 (95% CI = 1.28–2.45), respectively), with clinically relevant depressive symptoms. Notably, social participation has a 3.12% reduction from the logistic regression adjusted for only age and sex, peer-to-peer support decreases by 16.13%, supervisor support decreases by 40.82%, and having a partner decreases by 20.41%, while WLI increases by 11.32% (Table 3).

Furthermore, we conducted a moderation analysis using sex as a moderator. Except for one moderation analysis, all others were found to be nonsignificant. Specifically, supervisor support is a protective factor for females (OR = 0.52; *p* < 0.001) while acting as a risk factor for depressive symptoms in males (OR = 1.77; *p* = 0.010). Detailed results are shown in Table 4.

**Figure 1:**
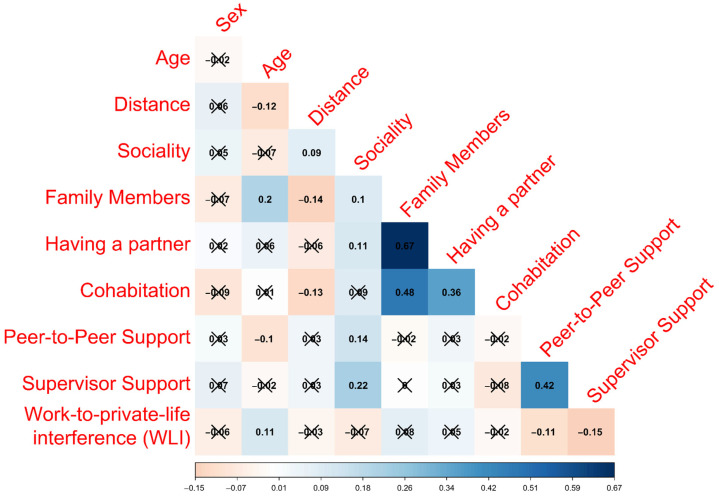
Multicollinearity model analysis.

The multicollinearity analysis revealed no collinearity (Kendall’s τ < 0.50) among the independent variables except for the pair “family members” and “cohabitation” (Figure 1).

## 4. Discussion

This study aims to assess the associations between the social network characteristics of Italian PHRs and depressive symptoms. In our analyses, with a primary focus on clinically relevant depressive symptoms (PHQ-9 ≥ 10), high social participation and having a partner emerge as protective factors, reducing the risk by 61% and 41%, respectively. Similarly, a supportive work environment with high peer-to-peer and supervisor support reduces the risk by 28% and 31%, respectively. On the other hand, high work-to-private life interference is associated with a 59% increased risk of clinically relevant depressive symptoms. The adoption of two cut-offs for PHQ-9, one for assessing mild-to-moderate depressive symptoms (PHQ-9 ≥ 5) and the other for clinically relevant depressive symptoms (PHQ-9 ≥ 10), allows for a more sensitive approach to identifying potential risks or protective factors. In our case, the results are comparable for both cut-offs, with the same variables showing statistically significant associations with the outcome, except that having a partner is significantly associated only with clinically relevant depressive symptoms. Similarly, when considering PHQ-9 as a continuous score, the results remain consistent. Moreover, to enhance this study’s internal consistency, a multicollinearity analysis was performed. The absence of a strong correlation between almost all the variables indicates that they measure different characteristics of the study population’s social network. The only notable correlation was found between family members and cohabitation (τ = 0.68), likely because family members are often part of the cohabiting group.

Our results are aligned with the scientific literature. Indeed, it has long been recognized that social support is strongly associated with mental health [47]. As noted by Holt-Lundstad et al. (2010), robust social relationships can significantly extend survival by 50%, while, conversely, poor social relationships may be more detrimental than excessive alcohol consumption, smoking, obesity, and lack of exercise [48]. Similarly, the role of social networks in mental health has been extensively studied, with reports consistently indicating that individuals with smaller networks, fewer interpersonal relationships, or lower levels of social support tend to have high rates of depression [49,50].

Among functional characteristics of the social network, both peer-to-peer and supervisor support were found to be protective against mild-to-severe and clinically relevant depressive symptoms, while work-to-private life interference emerged as a risk factor. A recent meta-analysis found that emotional forms of support are more closely associated with depression than instrumental forms of support, especially among adults aged 18–50 [51]. Regarding peer-to-peer support, the literature provides examples of its possible utility in reducing mental health symptoms. Interventions aimed at improving mental health in young people have demonstrated that peer-to-peer support effectively reduces negative effects [52]. Other studies have shown its positiveness on happiness, self-esteem effective coping, and reductions in depression, loneliness, and anxiety [53]. Furthermore, gender differences in this context are emphasized in the literature. Globally, females have a higher risk of suffering from depression and related symptoms than males [54,55], a situation that was exacerbated during the COVID-19 pandemic, possibly because females were more likely to be affected by its social consequences [56]. In light of these findings, we not only adjusted our regression models by sex but also conducted a moderation analysis to explore the relationship among each variable and clinically relevant depressive symptoms across different sexes. Supervisor support was found to be a risk factor for depressive symptoms in males, while it was protective for females. The positive association between supervisor support and healthcare workers’ mental well-being is supported by the literature [57,58]. Low supervisor support has also been shown to amply the association between social stressors and depressive symptoms [59]. However, when considering gender differences, supervisor support has been demonstrated to be a protective factor for work-related stress in females but not in males [60]. According to a recent cohort study, low supervisor support is a risk factor for depression in females but not in males [61]. These results can be interpreted in light of the masculine norms theory, which suggests that masculine norms reduce males’ willingness to seek and accept help during periods of psychological distress [62]. Therefore, it is possible that among our sample of male PHRs, a supportive supervisor could be perceived as a threat to their masculinity.

A high work-to-private life interference has previously been demonstrated as a predictive factor not only for depression but also for treatment in the general population [63]. In a longitudinal study, it was found that only females with high work-to-private-life interference were significantly more likely to develop major depression [64]. However, these specific results were not observed in our analyses. Despite the increasing focus on family friendly policies aimed at ensuring gender equality in Western countries, household tasks and responsibilities are still predominantly shouldered by females [65]. This suggests that a high work-to-private life interference may be more closely associated with females’ lives and their mental health than males. Nevertheless, it should be noted that our study population consisted of young adults who have not yet started a family, which could explain the absence of a gender difference in this association.

Among the structural characteristics of the social network in our study, having a partner was found to be a protective factor. This finding aligns with other studies investigating mental health in young adults, the age group to which most of the PHRs belong [66,67,68]. However, the literature also reports that depressive symptoms and romantic relationships are connected in a bidirectional manner: fewer positive partner interactions, such as intimacy and support, and more negative partner interactions, such as conflicts, are associated with increased depressive symptoms [69,70]. Conversely, depressive symptoms can impact romantic relationships by generating stress and potentially influencing interpersonal behavior and choices, which can, in turn, lead to the end of the relationship [71,72,73]. The cross-sectional design of our study does not allow us to delve deeper into whether these considerations are applicable to our study population. In this context, further investigations are needed to confirm these findings.

Similarly to romantic relationships, social participation has been observed to both influence and be influenced by good mental health status [74,75]. Leading a meaningful life with a sense of purpose has been identified as a determinant of health and is associated with a reduced risk of depression [76]. Our study’s findings are in line with the existing literature.

### 4.1. Implication and Practices for Public Health Policies

The medical residency period is a crucial phase in a physician’s professional journey. Medical residents are tasked with both practical responsibilities and deepening their theoretical knowledge, often without the immediate support of colleagues or superiors. In addition, the high workload and job responsibilities frequently encroach on their family, social, or personal life, which constitutes their social network. It is important to note that medical residents, as a group, are more likely to experience depressive symptoms than the general population, due to several factors, as reported in the literature [77]. This is especially true for Italian PHRs, who exhibit a high prevalence of depressive symptoms, similar to what is observed in healthcare workers in general [78,79].

Our findings are particularly relevant as they offer insight into the distribution of depressive symptoms among Italian PHRs, a population that has received limited attention in prior research. Moreover, while much of the existing research focuses on specific determinants of the social network, our study explores the association between several social network characteristics and depressive symptoms. This comprehensive assessment suggests the possible use of our data in terms of managing and organizing residency courses. In general, PHRs usually spend a significant portion of their residency in close contact with their supervisor and colleagues, but specific contextual factors also play a crucial role. For instance, in Italy, the high rate of retirements and a simultaneous low hiring rate in the healthcare system have led to a widespread shortage of personnel. The COVID-19 pandemic has underscored the urgent need to bolster the national healthcare system, resulting in an increase in scholarships for medical residents. However, this has led to fewer supervisors available for a larger number of PHRs enrolled in residency programs.

In light of this, the combination of external stressors, such as the COVID-19 pandemic, and the limited professional support available to PHRs may have contributed to the rising prevalence of depressive symptoms. Therefore, our findings can aid and inform policymakers in the management of residency training programs.

Furthermore, our results highlight the importance of applying a gender perspective in mental health studies to gain a proper understanding of the phenomenon and subsequently implement effective and proportionate interventions. Lastly, our study strengthens the need to explore gender differences in various mental health conditions among PHRs.

### 4.2. Limitations and Strengths

Before generalizing our results, the limitations and strengths of our work should be considered. Firstly, this is a cross-sectional study, which, by definition, makes it impossible to measure incidence or investigate the temporal relationship between exposures and outcomes. In a cross-sectional design, outcomes and exposures are measured simultaneously, and causality cannot be inferred. Particularly in this study, it is difficult to establish whether the absence of a supportive social network is a risk factor or a consequence of depressive symptoms. This is because depression often profoundly affects all aspects of life, and individuals with depression tend to have fewer rewarding and more dysfunctional social relationships compared with others [80]. Furthermore, participation in this study was voluntary, which could potentially introduce a selection bias. It is possible that individuals with depressive symptoms or those who are more socially isolated may be less prone to participate. Therefore, this type of potential selection bias may have resulted in an underestimation of the strength of the associations. Moreover, this study used self-reported measures, and people might have answered in accordance with social norms rather than truthfully [81]. Despite the precautions put in place, the results might be prone to social desirability bias [82].

Nevertheless, our study also has some strengths. It is a nationwide survey with a good representativeness of Italian PHRs [78]. In addition, it used a rigorous methodology that enhanced the internal consistency of the results. Furthermore, the role of gender was explored, considering the well-known influence of gender on mental health and healthcare accessibility [83,84,85]. Moreover, in terms of statistical analysis, we used advanced statistical techniques, considering depression as both a continuous and a dichotomous variable, and conducting linear and multiple logistic regression analyses. Moreover, we adjusted the model not only for sex and age but also for physical activity and alcohol. Lastly, to increase the robustness of our results, we conducted multicollinearity and moderation analyses. Across all these analyses, the main results remained consistent and did not materially change.

## 5. Conclusions

Our data contribute to building an evidence base of social network characteristics and their association with depressive symptoms among medical residents. Among the functional aspects of the social network, peer-to-peer and supervisor support are the most strongly associated with clinically relevant depressive symptoms, whereas, for the structural aspects, having a partner and social participation showed a protective role. Future research could delve into how the distribution of these social network characteristics persists and how they are associated with mental health, not only among medical residents but also among attending physicians and specialized medical doctors in general. In conclusion, our findings are valuable in understanding factors associated with depression and can contribute to reducing the high burden of mental health. These insights can inform policymakers and school directors in the planning and management of the training and professional activities of medical residents.

## Figures and Tables

**Table 1:** Relationships between socio-demographic characteristics and mild-to-severe (PHQ ≥ 5) and clinically relevant depressive symptoms (PHQ-9 ≥ 10).

Characteristic	Overall	PHQ-9 Score ≥ 5			PHQ-9 Score ≥ 10		
	n = 379	No (n = 148)	Yes (n = 231)	*p*	No (n = 282)	Yes (n = 97)	*p*
Sex				0.453 ^1^			0.802 ^1^
Female	219 (57.78%)	82 (55.41%)	137 (59.31%)		164 (58.16%)	55 (56.70%)	
Male	160 (42.22%)	66 (44.59%)	94 (40.69%)		118 (41.84%)	42 (43.30%)	
Age	30.00 (29.00, 34.00)	30.00 (28.00, 34.00)	31.00 (29.00, 33.00)	0.446 ^2^	30.00 (28.00, 34.00)	31.00 (29.00, 33.00)	0.134 ^2^
Functional characteristics of social network							
Peer-to-Peer Support	4.00 (3.00, 5.00)	4.00 (4.00, 5.00)	4.00 (3.00, 5.00)	<0.001 ^2^	4.00 (4.00, 5.00)	4.00 (3.00, 4.00)	0.031 ^2^
Supervisor Support	4.00 (3.00, 4.00)	4.00 (3.00, 5.00)	3.00 (3.00, 4.00)	<0.001 ^2^	4.00 (3.00, 4.00)	3.00 (2.00, 4.00)	<0.001 ^2^
WLI	3.00 (2.00, 3.00)	2.00 (2.00, 3.00)	3.00 (2.00, 3.00)	<0.001 ^2^	2.00 (2.00, 3.00)	3.00 (2.00, 4.00)	<0.001 ^2^
Structural characteristics of social network							
Distance	0.00 (0.00, 122.29.0)	0.00 (0.00, 201.27)	0.00 (0.00, 0.00)	0.026 ^2^	0.00 (0.00, 137.52)	0.00 (0.00, 111.21)	0.786 ^2^
Family Members	2.00 (2.00, 2.00)	2.00 (2.00, 2.00)	2.00 (2.00, 2.00)	0.870 ^2^	2.00 (2.00, 2.00)	2.00 (2.00, 2.00)	0.124 ^2^
Having a partner				0.141 ^1^			0.043 ^1^
Yes	276 (72.82%)	114 (77.03%)	162 (70.13%)		213 (75.53%)	34 (35.05%)	
No	103 (27.18%)	34 (22.97%	69 (29.87%)		69 (24.47%)	63 (64.95%)	
Cohabitation				0.760 ^1^			0.433 ^1^
Living Alone	98 (25.86%)	37 (25.00%)	61 (26.41%)		70 (25.0%)	28 (29.0%)	
With Others	281 (74.14%)	111 (75.00%)	170 (73.59%)		212 (75.0%)	69 (71.0%)	
Social participation	3.00 (2.00, 3.00)	3.00 (3.00, 4.00)	2.00 (1.00, 3.00)	<0.001 ^2^	3.0 (2.0, 4.0)	1.0 (1.0, 2.0)	<0.001 ^2^

PHQ-9: 9-item Patient Health Questionnaire; WLI: work-to-private life interference; ^1^ Pearson’s chi-squared test; ^2^ Wilcoxon rank sum test.

**Table 2:** Association between functional and structural characteristics of social networks and depressive symptoms calculated using multivariate linear regression models adjusted for age and sex.

Characteristic	aβ	95% CI ^1^	*p*
Functional characteristics of social network			
Peer-to-peer support	−1.01	−1.52; −0.50	<0.001
Supervisor support	−1.20	−1.67; −0.74	<0.001
Work-to-private life interference (WLI)	1.24	0.74; 1.74	<0.001
Structural characteristics of social network			
Distance	0.00	0.00; 0.00	0.636
Family members	−0.81	−1.54; −0.08	0.031
Having a partner (ref. = No)	−1.42	−2.58; −0.25	0.017
Cohabitation (ref. = Alone)	−1.13	−2.32; 0.06	0.062
Social participation	−2.13	−2.49; −1.76	<0.001

aβ: adjusted beta; CI: confidence interval; ^1^ Adjustment: age and sex.

**Table 3:** Logistic regression models assessing the association between social network characteristics and clinically relevant depressive symptoms (PHQ-9 ≥ 10).

	PHQ-9 Score ≥ 10			PHQ-9 Score ≥ 10 Sensitivity Analysis		
Characteristic	aOR ^1^	95% CI	*p*	aOR ^2^	95% CI	*p*
Functional characteristics of social network						
Peer-to-peer support	0.72	0.58; 0.90	0.004	0.62	0.47; 0.83	0.001
Supervisor support	0.69	0.55; 0.85	<0.001	0.49	0.36; 0.68	<0.001
Work-to-private-life interference (WLI)	1.59	1.25; 2.01	<0.001	1.77	1.28; 2.45	<0.001
Structural characteristics of social network						
Distance	1.00	1.00; 1.00	0.895	1.00	1.00; 1.00	0.816
Family Members	0.73	0.51; 1.03	0.075	0.73	0.51; 1.05	0.089
Having a partner (ref. = No)	0.59	0.36; 0.97	0.039	0.49	0.25; 0.96	0.036
Cohabitation (ref. = Alone)	0.82	0.49; 1.37	0.443	0.82	0.48; 1.40	0.466
Social participation	0.39	0.31; 0.50	<0.001	0.38	0.29; 0.49	<0.001

aOR: adjusted odds ratio; ^1^ Adjustment: age and sex; ^2^ Adjustment: age, sex, drinking risk, and physical activity.

**Table 4:** Relationships between socio-demographic characteristics and depression symptoms and moderation by sex.

Characteristic	Moderator	PHQ Score ≥ 10		
		aOR ^1^	95% CI	*p*
Functional characteristics of social network				
Peer-to-peer support	Predictor	0.63	0.48; 0.84	0.002
	Interaction	1.42	0.89; 2.27	0.142
Supervisor support	Predictor	0.52	0.38; 0.71	<0.001
	Interaction	1.77	1.11; 2.74	0.010
Work-to-private-life interference (WLI)	Predictor	1.74	1.27; 2.38	<0.001
	Interaction	0.81	0.50; 1.30	0.375
Structural characteristics of social network				
Distance	Predictor	1.00	1.00; 1.00	0.757
	Interaction	1.00	1.00; 1.00	0.761
Family Members	Predictor	0.80	0.55; 1.16	0.239
	Interaction	1.02	0.55; 1.88	0.959
Having a partner (ref. = No)	Predictor	0.47	0.25; 0.90	0.023
	Interaction	1.74	0.63; 4.86	0.289
Cohabitation (ref. = Alone)	Predictor	0.69	0.34; 1.40	0.310
	Interaction	1.42	0.50; 4.00	0.513
Social participation	Predictor	0.37	0.26; 0.51	<0.001
	Interaction	1.16	0.71; 1.90	0.555

^1^ Adjustment: age and sex.

## Data Availability

The authors can be contacted for information about the data presented.

## Supplementary Materials

The following supporting information can be downloaded at: https://www.mdpi.com/article/10.3390/bs13110881/s1, Table S1: Cronbach’s α values for each adopted questionnaire derived from the literature and estimated in the current study. Table S2: Descriptive statistics (mean, SD, skewness, kurtosis) of continuous variables; Figure S1: Distribution of continuous variables; Table S3: Correlation matrix of the predictor variables. Kendall’s tau correlation coefficient test was used; Table S4: Logistic regressions assessing the association between social network characteristics and mild-to-severe depressive symptoms.
