# Supplementary material for: Social Network Characteristics and Depressive Symptoms of Italian Public Health Medical Residents: The Public Health Residents’ Anonymous Survey in Italy (PHRASI)
Source: Behav Sci (Basel). 2023 Oct 25;13(11):881. doi: 10.3390/bs13110881 (PMC10798373; doi:10.3390/bs13110881)
Supplement: Supplementary file 1 [file behavsci-13-00881-s001.zip › behavsci-2564156-supplementary.pdf]

Supplementary Table S1. Cronbach's α values for each adopted questionnaire derived from literature and estimated in the current study.

Questionnaire	Acronym	Cronbach's α from original valitation study	Cronbach's α from current study
Patient Health Questionnaire -9	PHQ-9	0.89	0.88
Alcohol Use Disorders Identification Test	AUDIT-C	0.80	0.63
International Physical Activity Questionnaire	IPAQ	0.67	0.66
WHO-5 wellbeing index	WHO-5	0.86	0.85
Work Related Stress Questionnaire	WRSQ	0.88	0.80

Supplementary Table S2. Descriptive statistics (mean, SD, skewness, kurtosis) of continuous variables.

Variable	Mean	Standard Deviation	Skewness	Kurtosis	Standard Error
**Age**	31.61	4.49	1.54	3.58	0.23
**Distance**	120.83	245.07	2.19	3.99	12.59
**Sociality**	2.58	1.24	-0.09	-0.75	0.06
**Peer-to-Peer Support**	3.90	1.01	-0.95	0.58	0.05
**Supervisor Support**	3.51	1.08	-0.56	-0.35	0.06
**Work-to-private-life interference**	2.66	1.02	0.21	-0.46	0.05

Supplementary Figure S1. Distribution of continuous variables.

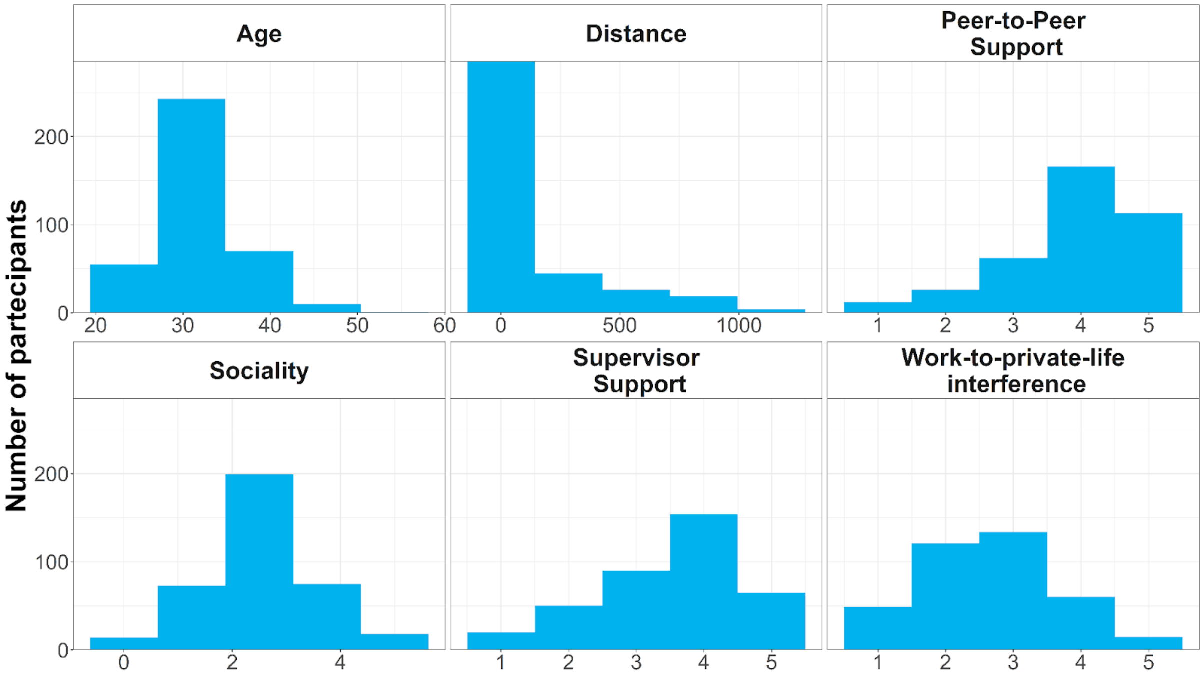

Supplementary Table S3. Correlation matrix of the predictor variables. Kendall's tau correlation coefficient test was used

	Sex	Age	Distance	Social participation	Family Members	Having a partner	Cohabitation	Peer-to-Peer Support	Supervisor Support	Work-to-private-life interference (WLI)
**Sex**	1.00	-0.02	0.06	0.05	-0.07	0.02	-0.09	0.03	0.07	-0.06
**Age**	-0.02	1.00	-0.12	-0.07	0.20	0.06	0.01	-0.10	-0.02	0.11
**Distance**	0.06	-0.12	1.00	0.09	-0.14	-0.06	-0.13	0.03	0.03	-0.03
**Social participation**	0.05	-0.07	0.09	1.00	0.10	0.11	0.09	0.14	0.22	-0.07
**Family Members**	-0.07	0.20	-0.14	0.10	1.00	0.67	0.48	-0.02	0.00	0.08
**Having a partner**	0.02	0.06	-0.06	0.11	0.67	1.00	0.36	0.03	0.03	0.05
**Cohabitation**	-0.09	0.01	-0.13	0.09	0.48	0.36	1.00	-0.02	-0.08	-0.02
**Peer-to-Peer Support**	0.03	-0.10	0.03	0.14	-0.02	0.03	-0.02	1.00	0.42	-0.11
**Supervisor Support**	0.07	-0.02	0.03	0.22	0.00	0.03	-0.08	0.42	1.00	-0.15
**Work-to-private-life interference (WLI)**	-0.06	0.11	-0.03	-0.07	0.08	0.05	-0.02	-0.11	-0.15	1.00

Supplementary Table S4. Logistic regressions assessing the association between social network characteristics and mild to severe depressive symptoms.

	**PHQ score ≥ 5**		
**Characteristic**	**OR**	**95% CI**	**p**
Functional characteristics of social network			
**Peer-to-Peer Support**	**0.64**	**0.50; 0.81**	**<0.001**
**Supervisor Support**	**0.62**	**0.50; 0.76**	**<0.001**
**Work-to-private-life interference (WLI)**	**1.64**	**1.31; 2.04**	**<0.001**
Structural characteristics of social network			
**Distance**	1.00	1.00; 1.00	0.059
**Family Members**	1.02	0.76; 1.37	0.880
**Having a partner (ref. = No)**	**0.70**	**0.43; 1.12**	**0.139**
**Cohabitation (ref. = Alone)**	0.91	0.57; 1.47	0.706
**Social participation**	**0.44**	**0.35; 0.54**	**<0.001**
